# The Relationship Between Emotional Intelligence and Diabetes Management: A Systematic Review

**DOI:** 10.3389/fpsyg.2021.754362

**Published:** 2021-11-04

**Authors:** Aida Pérez-Fernández, Pablo Fernández-Berrocal, María José Gutiérrez-Cobo

**Affiliations:** ^1^Department of Basic Psychology, Faculty of Psychology, University of Málaga, Málaga, Spain; ^2^Department of Developmental and Educational Psychology, Faculty of Psychology, University of Málaga, Málaga, Spain

**Keywords:** emotional intelligence, Type 1 diabetes, Type 2 diabetes, HbA1c, diabetes management, systematic review

## Abstract

Diabetes has been associated with affective disorders which complicate the management of the disease. Emotional intelligence (EI), or the ability to perceive, facilitate, understand, and regulate emotions, has shown to be a protective factor of emotional disorders in general population. The main objective of this study was to systematically review the role of the EI construct in Type 1 and Type 2 diabetics and to observe how EI is related to biological and psychological variables. Comprehensive searches were conducted in PubMed, Scopus, PsycInfo, and Cochrane without time limitations, for studies examining the link between diabetes and EI. A total of 12 eligible studies were selected according to the inclusion criteria. We divided the results into four sections: (1) EI and hemoglobin glycosylated (HbA1c), (2) EI training effects, (3) differences in EI between persons with diabetes and without diabetes, and (4) EI and psychological adjustment and well-being. The results showed negative correlations between EI and HbA1c, positive effects of EI training on quality of life, anxiety, and glycemic control, no differences in EI between people with diabetes and healthy individuals, and, finally, negative correlations between EI and different psychological variables such as diabetes-related anxiety and distress, and positive correlations with quality of life, well-being, and marital satisfaction. This systematic review offers a starting point for a theoretical and practical understanding of the role played by EI in the management of diabetes and reveals that EI is a promising protective factor for biological and psychological variables in this population.

## Introduction

Diabetes mellitus is a chronic disease associated with significant morbidity and mortality throughout the world (Wild et al., 2004). The global prevalence of diabetes in 2019 is estimated to be 9.3% (463 million people), which may increase to 10.2% (578 million) by 2030 and 10.9% (700 million) by 2045 (Saeedi et al., 2019). Diabetes is a condition in which the body does not properly metabolize glucose. Two types of diabetes can be distinguished: Type 1 or insulin-dependent diabetes and Type 2 diabetes. For Type 1 diabetes, the causes are still unknown, it is a chronic disease, and its onset has a higher incidence in young people and children. Type 2 diabetes is triggered by excess body weight and physical inactivity, and onset is usually in adulthood. People with Type 1 diabetes need insulin, while those with Type 2 diabetes can be treated with oral medications, exercise, and diet (WHO, 2020). Over time, high glucose levels can lead to severe consequences, such as stroke, heart attacks, blindness, and kidney failure. The primary purpose of diabetes care should be to keep glucose levels within healthy limits. The daily behaviors aimed at maintaining healthy glucose levels and preventing the severe consequences of the disease (especially in Type 1 diabetes), and all make this condition highly stressful. This routine puts people with diabetes at a higher risk of psychological problems (Musselman et al., 2003; Egede, 2004; Goldney et al., 2004). In turn, it has been observed that alterations in glucose variability in diabetics have an impact on mood (Gonder-Frederick et al., 1989; Hermanns et al., 2003). Additionally, several studies analyzing the neuropsychobiological mechanisms underlying this relationship have found that the increase in hormones related to depression and stress (Sapolsky et al., 2000; Goodnick, 2001) also increases glucose levels.

Specifically, diabetes is associated with an increased risk of affective disorders in the adult population (Hislop et al., 2008; Gendelman et al., 2009). The relationship between diabetes and problems such as anxiety, depression, and anguish related to diabetes is attracting more attention due to its high prevalence (Anderson et al., 2001; Grigsby et al., 2002; Nicolucci et al., 2013) and its negative effect on self-care, glycemic control, and risk of complications and mortality (Collins et al., 2009; Park et al., 2013; Smith et al., 2013). Importantly, these disorders are associated with fewer self-care behaviors, poorer clinical outcomes, lower quality of life, and more severe and earlier onset of diabetes (Peyrot et al., 1999; Sipkoff, 2005; Gendelman et al., 2009; Lin et al., 2010). Consequently, psychology plays an essential role in the management of this disease, particularly when dealing with the practical problems and day-to-day diabetes routine. Due to this, it would be of interest to look for protective factors for population with diabetes that favor better control of the disease. One factor that researchers have recently been paying attention to is the emotional intelligence (EI) ability. Mayer and Salovey (1997) have defined this construct as:

The ability to perceive accurately, appraise, and express emotion; the ability to access and generate feelings when they facilitate thought; the ability to understand emotion and emotional knowledge; and regulate emotions to promote emotional and intellectual growth (p. 10).

Emotional intelligence has been conceptualized into three models depending on the measurement instrument used and the conceptualization of the construct: performance-based ability model, self-report ability models, and self-report mixed models (Joseph and Newman, 2010). The performance-based ability models evaluate EI through performance tests in an objective manner and conceive EI as a set of emotional skills based on the definition of Mayer and Salovey (1997). These models have greater empirical support (Mayer et al., 2016) than the other models. The self-report ability models are also based on these authors’ conceptualization, although they use subjective self-report measures. Finally, although they use self-report instruments, the self-report mixed models include a broader number of variables to define EI, such as mental abilities, personality factors, motivations, interpersonal and intrapersonal skills, and other facets (Bar-On, 2004). It is crucial to consider the model employed as the previous literature suggests there are weak correlations among them, suggesting that the models do not cover the same construct (Goldenberg et al., 2006).

The existing literature has shown that people with higher EI scores are more readily able to cope with stressors and problems in daily life, have closer relationships, and more significant social support networks (Zeidner et al., 2012). Consequently, EI is perceived as an indicator of psychological adjustment and is associated with well-being (Sánchez-Álvarez et al., 2016), happiness, and life satisfaction (Zeidner et al., 2012). In addition, there are also other reasons for proposing the hypothesis that EI is a critical element of diabetes. A higher EI level has been linked to healthier behaviors (Fernández-Abascal and Martín-Díaz, 2015), better health (Mikolajczak et al., 2015), along with fewer negative emotions such as anxiety (Killgore et al., 2016), depression (Fernández-Berrocal and Extremera, 2016), or distress in the face of adversity (Armstrong et al., 2011). In clinical populations, the level of EI is lower among people with inflammatory disease (such as rheumatoid arthritis, ankylosing spondylitis, or multiple sclerosis) compared to the healthy population (Costa et al., 2014).

### Rationale

Considering the previous literature, the primary purpose of this study is to systematically review the role of the EI construct in people with Type 1 and Type 2 diabetes by observing how EI is related to both biological and psychological variables in this population. We hypothesized that higher levels of EI would be a protective factor of diabetes management, as shown by better glycemic control and higher levels of psychological adjustment and well-being.

## Materials and Methods

### Literature Search

PubMed, Scopus, PsycInfo, and Cochrane databases were searched exhaustively, without time limitations, for studies examining the link between diabetes and emotional intelligence. Searches were conducted using the following keywords in English: “Diabetes” combined with “emotional intelligence” as terms in the title or abstract. The searches were undertaken between September 2020 and September 2021.

### Inclusion Criteria

To be included in the review, papers had to meet the following criteria: (1) empirical research providing data on the relationship between EI with any biological or psychological variable related to diabetes, (2) a sample with a Type 1 or Type 2 diabetes diagnosis, (3) a sample of any ethnicity, gender, or age (4) use of a valid and reliable emotional intelligence scale, and (5) articles written in Spanish or English.

### Exclusion Criteria

Letters, theses, comments, editorials, reports, or book chapters on previously published studies, intervention protocols, qualitative studies, and non-English or non-Spanish language publications were excluded.

### Data Extraction

**Figure 1:**
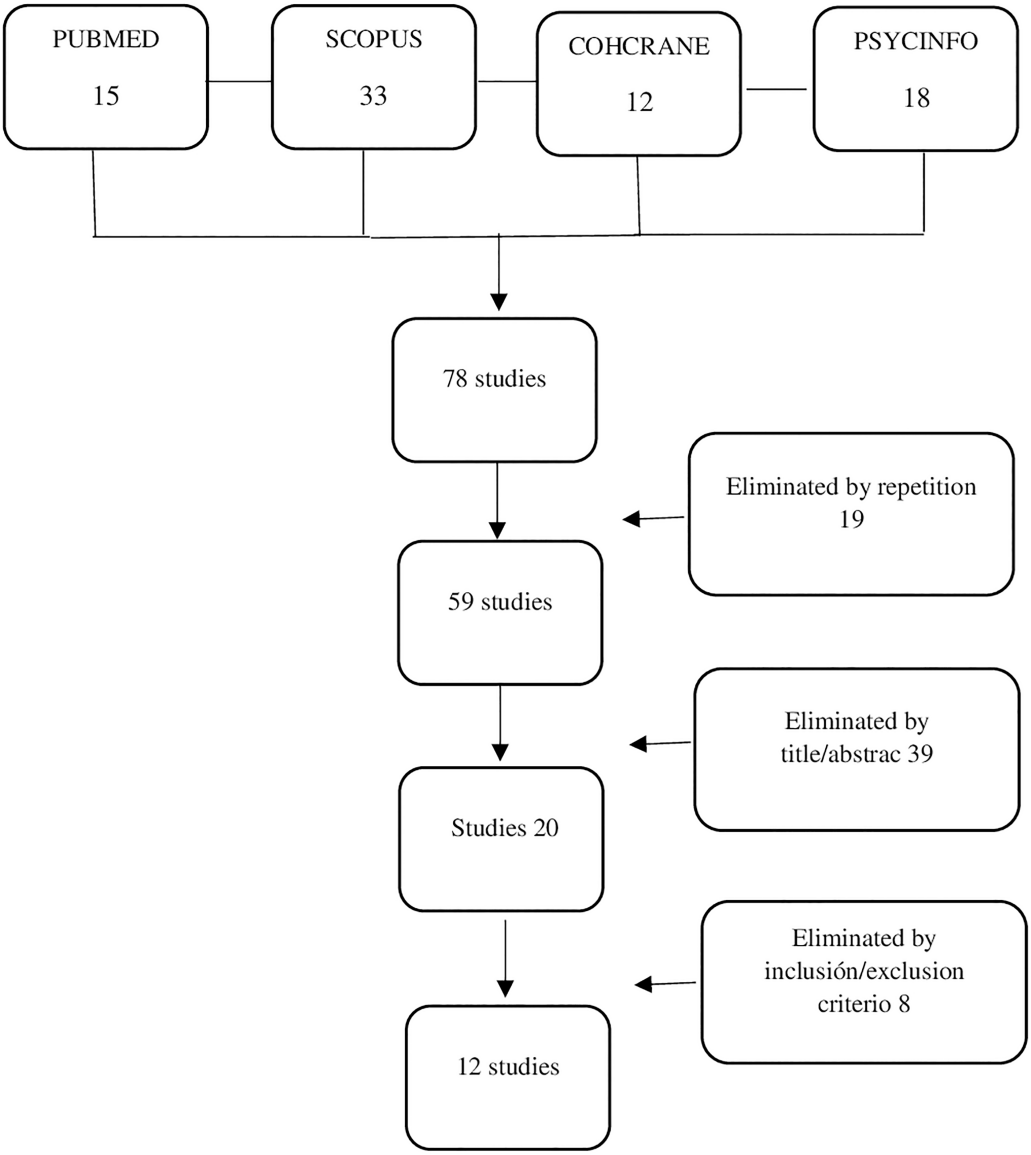
Prisma flow diagram for the selection of the literature included in this study.

The initial database search identified 78 potentially eligible studies: 33 from Scopus, 15 from PubMed, 12 from Cochrane, and 18 from PsycInfo. After removing duplicates, this resulted in 59 studies. Two reviewers independently assessed the titles and abstracts of all of the reports identified. Of these 59 studies, only 20 were selected for full text review after considering the inclusion/exclusion criteria specified, and 12 studies were finally included. Disagreements were resolved by discussion with the senior reviewer. The process of finding and selecting the items is shown in Figure 1. In order to analyze the quality of the studies, we have included a table of quality assessment using the Mixed Methods Appraisal Tool (MMAT; Hong et al., 2018; Table 1).

**Table 1:** Quality assessment of the included studies using the Mixed Methods Appraisal Tool (MMAT).

Quantitative non-randomized studies	Zysberg et al., 2017	Ruiz-Aranda et al., 2018	Hughes et al., 2012	Zysberg et al., 2013	Coccaro et al., 2016	Moradi et al., 2020	Žilinskiene et al., 2021	Schinckus et al., 2018	Dehghan et al., 2014
S1. Are there clear research questions?	Yes	Yes	Yes	Yes	Yes	Yes	Yes	Yes	Yes
S2. Do the collected data allow to address the research questions?	Yes	Yes	Yes	Yes	Yes	Yes	Yes	Yes	Yes
3.1. Are the participants representative of the target population?	Yes	Cannot tell	Yes	Yes	Yes	Yes	Yes	Yes	Yes
3.2. Are measurements appropriate regarding both the outcome and intervention (or exposure)?	Yes	Yes	Yes	Yes	Yes	Yes	Yes	Yes	Yes
3.3. Are there complete outcome data?	Yes	Yes	No	Yes	Yes	Yes	Yes	No	Yes
3.4. Are the confounders accounted for in the design and analysis?	Yes	Yes	Yes	Yes	Yes	Yes	Yes	No	Yes
3.5. During the study period, is the intervention administered (or exposure occurred) as intended?	Yes	Yes	Yes	Yes	Yes	Yes	Yes	Yes	Yes
Quantitative randomized controlled trials	Tavakol et al., 2018			Yalcin et al., 2008			Karahan and Yalcin, 2009		
S1. Are there clear research questions?	Yes			Yes			Yes		
S2. Do the collected data allow to address the research questions?	Yes			Yes			Yes		
2.1. Is randomization appropriately performed?	Yes			Yes			Yes		
2.2. Are the groups comparable at baseline?	Yes			Yes			Yes		
2.3. Are there complete outcome data?	Yes			Yes			Yes		
2.4. Are outcome assessors blinded to the intervention provided?	Cannot tell			Cannot tell			Cannot tell		
2.5. Did the participants adhere to the assigned intervention?	Yes			Yes			Yes		

### EI Instruments

We next define the instruments used to measure EI in the studies selected.

- The audiovisual emotional intelligence test (AVEI; Zysberg et al., 2011). This questionnaire belongs to the performance-based ability model of EI and measures the ability to perceive emotions. Twenty-seven audiovisual items are presented to the respondents (half are video clips and the other half images), and the participants must identify the emotion experienced by people portrayed in various personal and interpersonal settings. The total score measures correct responses, so a high score represents a higher level of EI. The reliability of the test has been shown to be acceptable (ranging from 0.67 to 0.78 in various settings).
- The Emotional Processing subscale of the Emotional Approach Coping Scale (Stanton et al., 2000) belongs to the self-report ability models of EI. The scale consists of four items that are scored on a Likert scale that ranges from 1 (never) to 5 (always). It measures how the respondents recognize, accept, and understand their own emotions. The internal consistency and test–retest reliability of the scale were *α*=0.72.
- The Schutte Self-Report Emotional Intelligence Test (SEIS; Schutte et al., 1998) belongs to the self-report ability models of EI. This consists of 33 items that are scored on a Likert-type scale that ranges from 1 (strongly agree) to 5 (strongly disagree) in which respondents indicate their level of agreement with various statements on aspects of EI. It includes four subscales: emotion perception, utilizing emotions, managing self-relevant emotions, and managing others’ emotions. The scale presents a high reliability of 0.90.
- The Trait Meta Mood Scale (TMMS; Salovey et al., 1995) belongs to the self-report ability models of EI. It includes 30-items on a Likert scale ranging from 0 to 4 (from “totally disagree” to “totally agree”) and evaluates a total of three dimensions: “Attention” to emotions, which refers to people’s belief in how much they attend to their feelings; “clarity” of emotions that measures how a person thinks that they perceive emotions, and, finally, emotional “repair” which evaluates a person’s efforts to maintain positive emotions and block negative moods. Internal reliability for all three factors ranges from *α*=0.62 to 0.87.
- The short version of EI-DARL (Antinienė and Lekavičienė, 2014). This belongs to the self-report ability models of EI. It consists of 73 questions in which respondents must report their agreement or disagreement on a 6-point Likert scale. This scale evaluates a total of five dimensions: “Understanding your emotions,” “Your emotional control,” “Understanding emotions of the other person,” “Control of other emotions,” and “Manipulations.” The internal consistency was *α*=0.92 (Žilinskiene et al., 2021).
- BarOn Emotional Quotient Inventory (EQ-I; Bar-On, 2004, 2006). This belongs to the self-report mixed models of EI. It includes 133 items with a Likert response scale of 1–5 (ranging from very rarely or not true for me to very often true for me or true for me). The scale provides an estimate of emotional and social intelligence. It is made up of five composite scales and 15 subscales: intrapersonal scales (self-regard, emotional self-awareness, assertiveness, independence, self-esteem), interpersonal scales (empathy, social responsibility, interpersonal relationships), adaptability scales (reality tests, flexibility, problem-solving), stress management scales (stress tolerance, impulse control), and general scales of the state of mood (optimism, happiness). EQ-I has proven to be a consistent, stable, and reliable measure. Its overall internal consistency is *α*=0.97.
- The Shrink emotional intelligence questionnaire (Moradi et al., 2020). This belongs to self-report mixed models of EI. It consists of 33 items divided into five subscales (self-motivation, self-awareness, self-management, coherence, and social skills). Its reliability coefficient was as follows: self-motivation=54%, self-awareness=69%, self-management=64%, coherence (social intelligence)=51%, social skills=50%, and the global score=85%.
- The profile of emotional competence (PEC; Brasseur et al., 2013) belongs to the self-report mixed models of EI. The questionnaire comprises 50 items scored on a 1–5 scale (ranging from strongly disagree to strongly agree). The final result offers three global scores: an intrapersonal EC score (*α*=0.86) and interpersonal EC score (*α*=0.89) and a total EC score (*α*=0.92).
- The Trait Emotional Intelligence Questionnaire: Short Format (TEIQue – SF; Petrides, 2009) belongs to the EI self-report mixed models. It consists of 30 items and uses a 7-point scale. This questionnaire assesses four constructs: well-being, self-control, emotionality, and sociability. The internal consistency of the scale was good (*α*=0.881).

## Results

Emotional intelligence was measured with nine different questionnaires across the 12 selected articles. In addition, these instruments were associated with different variables related to diabetes, such as HbA1c, distress, and well-being. Given the variability in the studies, the results are divided into four main sections. The first section focuses on results concerning the relationship between EI and HbA1c. The second presents studies that evaluate how EI training affects biological and psychological diabetes variables. Third, we describe those articles that compare EI between persons with diabetes and without diabetes, and finally, we present those results that relate EI with measures of psychological adjustment and well-being that have not previously been described.

### EI and HbA1c

One of the main findings of this review concerns the relationship between EI and HbA1c level. HbA1c is an indicator of mean blood glucose over the past 2–3 months and is highly sensitive to changes in blood glucose levels (Kyngäs, 2000). It is the most common and widely acceptable indicator of long-term glycemic balance (Sato, 2014). Higher levels of HbA1c indicate inadequate glycemic control. We found six studies that directly evaluated the association between EI and HbA1c (Table 2). In adults, three studies found a negative association between EI and HbA1c (Coccaro et al., 2016; Zysberg et al., 2017; Ruiz-Aranda et al., 2018). This means that higher levels of EI were related to a lower level of HbA1c. Of these three studies, two were conducted with people diagnosed with Type 1 diabetes (Zysberg et al., 2017; Ruiz-Aranda et al., 2018).

**Table 2:** Studies analyzing the relationship between emotional intelligence (EI) and HbA1c levels.

Study	Design and objectives	Sample	Type of diabetes	EI Scale	Outcome measures	Results
Zysberg et al., 2017 Israel	Cross-sectional study To evaluate the hypothesis that EI will show negative associations with blood glucose and HbA1c level	78 young adults. 61.5% females (mean age 31.89±9.97years) No psychological or medical intervention described	Type 1 diabetes	The audio-visual test of emotional intelligence (AVEI) Performance-based ability model Mean=17.87±2.90	Blood levels of sugar/glucose during the last day HbA1c levels	A negative association between EI and HbA1c and marginal results in the same direction with blood sugar levels
Ruiz-Aranda et al., 2018 Israel	Cross-sectional study To examine the relationship between EI and HbA1c levels in a sample of patients with Type 1 diabetes	85 adults. 62% females (mean age 31±9.97years) No psychological or medical intervention described	Type 1 diabetes	The AVEI Performance-based ability model Mean=17.87±2.90	HbA1c level	EI showed a negative association with HbA1c
Hughes et al., 2012 United States	Cross-sectional study To examine whether emotional processing, self-control and the interaction between these variables predicted HbA1c for adolescents with Type 1 diabetes in addition to diabetes-specific constructs	137 adolescents. 54% females (mean age 13.48±1.51years) Approximately half (63%) of the adolescents were on an insulin pump, with the remainder prescribed MDI	Type 1 diabetes	The Emotional Approach Coping Scale. Self-report ability model Mean=11.81±3.02	HbA1c level Self-control Self-control scale (Finkenauer et al., 2005)	EI in interaction with self-control is negatively related to HbA1c
Zysberg et al., 2013 Israel	Cross-sectional study To evaluate the hypothesis that parents’ emotional intelligence is associated with their children’s Type I diabetes	81 parents. 54.3% females (mean age 41.12±6.90years). The mean age of the children was 9.9±3.41years No psychological or medical intervention described	Type 1 diabetes	The AVEI Performance-based ability model Mean=14.33±3.46 The Schutte Self-Report Emotional Intelligence Test (The SEIS) Self-report ability model Mean=2.00±0.44	HbA1c level	A negative relationship was found between EI of parents and the HbA1c of their children
Coccaro et al., 2016 United States	Cross-sectional study To investigate the relationship between measures of emotional regulation and EI and HbA1c levels in adult patients with Type 2 diabetes	100 adults. 55% females (mean age 59±13years) No psychological or medical intervention described	Type 2 diabetes	Trait Meta-Mood (TMMS) Self-report ability model Mean=78.8±11.0	HbA1c level	EI showed a negative association with HbA1c
Žilinskiene et al., 2021 Lithuania	Cross-sectional study To investigate the association between mothers’ EI and Type I diabetes disease management in their children	134 mothers (mean age 37.83±4.37years) 134 children 51.5% female (mean age 9.26±2.03years) No psychological or medical intervention described	Type 1 diabetes	the short version of EI-DARL Self-report ability model	HbA1c level	An increase in scores of the EI scales and subscales of mothers increases the likelihood of deterioration in T1DM management of their children

Regarding the adolescent population, two studies were found. Zysberg et al. (2013) analyzed how the parent’s EI was related to the HbA1c levels of their offspring, and they found a negative correlation between both variables. That is, the higher the parent’s EI, the lower the offspring’s HbA1c. The study by Hughes et al. (2012) also showed a negative relationship between the EI of young people and their HbA1c. Specifically, EI uniquely predicted variance in metabolic control above other diabetes-specific constructs measured in the study, such as self-control, self-efficacy, or adherence.

Finally, in a sample of 134 children and in contrast to previous results, Žilinskiene et al. (2021) found that a higher EI perception of mothers was related to poorer metabolic control in their children; that is, the ability of mothers to understand and regulate their own emotions, understand the causality of one’s own emotions, and transform one’s negative emotions into positive emotions did not facilitate the management of diabetes in their offspring.

In summary, in five out of six studies, higher levels of EI were related to better HbA1c. In the case of adults, they included individuals with Type 1 and 2 diabetics. Finally, regarding the effect of parental EI on the HbA1c of their offspring, EI appears to be beneficial for the glycemic control of an adolescent sample, while, contrary to expectations, counterproductive in a sample of children.

### EI Training and Diabetes

Three studies found a link between EI training and quality of life, well-being, anxiety, and HbA1c in samples of adults with Type 2 diabetes (Table 3). Tavakol et al. (2018) showed that the application of a self-care EI program improved the HbA1c levels of the participants, while Yalcin et al. (2008) showed that EI training improved quality of life and well-being in persons with diabetes and that these improvements persisted over time. Finally, Karahan and Yalcin (2009) evaluated the effect of EI training on the emotional burnout, anxiety, and HbA1c levels of persons with diabetes. The authors found that the program positively affected all these parameters compared to a control group of people with diabetes.

**Table 3:** Studies analyzing EI training in people with diabetes.

Study	Design and objectives	Sample	Type of diabetes	EI Scale	Outcome measures	Results
Tavakol et al., 2018 Iran	Randomized Controlled Trial To investigate the effect of self-care education on EI and HbA1c in patients with Type 2 diabetes	42 adults. 73.8% females. Control group *n*=21 (mean age 45.42±7.71years). Intervention group *n*=21 (mean age 48.57±7.89years) No medical intervention described	Type 2 diabetes	The BarOn questionnaire (EQ-I) Self-report mixed model Intervention before: Mean=65.09±6.49 Control before: Mean=64.68±7.66 Intervention after: Mean=70.95±6.92 Control after: Mean=64.38±7.09	HbA1c level	Self-care education improved HbA1c and EI levels
Yalcin et al., 2008 Turkey	Randomized Controlled Trial with 1st and 2nd follow-ups. To investigate the effect of an EI program on the health-related quality of life and well-being of individuals with Type 2 diabetes	36 adults. 50% females Study group *n*=18 (mean age 54.33±7.34years) Control group *n*=18 (mean age 51.17±5.81years) No medical intervention described	Type 2 diabetes	The BarOn questionnaire (EQ-I) Self-report mixed model Intervention before: Mean=97.77±8.98 Control before: Mean=98.38±8.89 Intervention after: Mean=124.27±5.64 Control after: Mean=98.88±9.31	HbA1c level Well-Being Well-Being Questionnaire (WHO-WBQ-22; Savli and Sevinc, 2005) Quality of Life WHO-Quality of Life (WHOQOL-Bref; Skevington et al., 2004)	EI training improved quality of life and well-being in persons with diabetes over time
Karahan and Yalcin, 2009 Turkey	Randomized Controlled Trial with 1st and 2nd follow-ups. To investigate the effects of an “EI Skills Training Program” on anxiety levels, burnout, and HbA1c in Type 2 diabetes mellitus patients	36 adults. 50% females Training group *n*=18 (mean age 53.06±4.43years) Control group *n*=18 (mean age 52.22±5.2years) No medical intervention described	Type 2 diabetes	The BarOn questionnaire (EQ-I) Self-report mixed model Intervention before: Mean=97.0±6.3 Control before: Mean=96.7±6.0 Intervention after: Mean=127.1±6.1 Control after: Mean=97.0±6.1	HbA1c level Anxiety Beck Anxiety Inventory (BAI; Beck et al., 1988) Burnout Maslach Burnout Inventory (MBI; Maslach and Jackson, 1981)	EI program improved emotional burnout, anxiety and HbA1c

Taken together, these studies showed the positive effect of EI training in improving biological and psychological factors in people with Type 2 diabetes.

### Differences Between People With Diabetes and Without Diabetes

Two studies have focused on comparing EI between persons with diabetes and without diabetes (Table 4). Schinckus et al. (2018) analyzed whether the levels of EI of people with diabetes (Type 1 and 2 indistinguishable) differed from those without diabetes. The results showed that the persons with diabetes in the study had lower intrapersonal and interpersonal EI, which resulted in a lower global emotional intelligence score than that of people without diabetes. However, when matching both groups in terms of gender, age, and educational level, the differences between the groups disappeared. Moradi et al. (2020) compared the EI and quality of life of elderly with diabetes (Type 1 and 2 indistinguishable) and without diabetes. The results showed no statistically significant differences in EI and quality of life between persons with diabetes and without diabetes.

**Table 4:** Studies analyzing differences between people with diabetes and without diabetes.

Study	Design and objectives	Sample	Type of diabetes	EI Scale	Outcome measures	Results
Schinckus et al., 2018 Belgium	Cross-sectional study To explore whether the levels of EI of people with diabetes differs from the non-diabetes population	Study 1: 8,532 participants (60.2% females) of which 333 had diabetes (mean age was 55.7±13.5years) Not psychological or medical intervention described	Type 1 and 2 diabetes	Study 1: The profile emotional competence (PEC) Self-report mixed model Diabetes group: Mean=3.37±0.42 Control group: Mean=3.47±0.43	Study 1: EI	The persons with diabetes in the study had a lower global EI score than that of people without diabetes. However, when matching both groups in terms of gender, age and educational level, the group differences disappeared
Moradi et al., 2020 Iran	Cross-sectional study To determine the effect of EI on the quality of life of elderly diabetic patients	People with diabetes *n*=63 (mean age 65.01±6.08years) and people without diabetes *n*=66. 47.3% females. No psychological or medical intervention described	Type 1 and 2 diabetes	The Shrink emotional intelligence questionnaire Self-report mixed model Diabetes group: Mean=99.42±10.37 Control group: Mean=97.18±18.49	EI	No statistically significant differences were found in EI between persons with diabetes and without diabetes

According to the two studies described, there were no differences between the population with diabetes (Type 1 and 2 indistinguishable) and without diabetes.

### Emotional Intelligence and Psychological Adjustment and Well-Being

Given the heterogeneity of the variables included in the studies selected for the review, the last section focused on the results of studies that explored the link between EI and other variables related to lifestyle of people with diabetes not previously mentioned (Table 5). Specifically, the results were divided into four sections: the relationship between EI and anxiety and burnout, EI and diabetes-related distress, EI and quality of life and well-being, and, finally, EI and marital satisfaction.

**Table 5:** Studies analyzing the relationship between EI and psychological adjustment and well-being.

Study	Design and objectives	Sample	Type of diabetes	EI Scale	Outcome measures	Results
Karahan and Yalcin, 2009 Turkey	Randomized Controlled Trial and Cross-sectional study To investigate the relationship between EI and anxiety levels and burnout in Type 2 diabetes patients	36 adults. 50% females Training group *n*=18 (mean age 53.06±4.43years) Control group *n*=18 (mean age 52.22±5.2years) No medical intervention described	Type 2 diabetes	The BarOn questionnaire (EQ-I) Self-report mixed model Intervention before: Mean=97.0±6.3 Control before: Mean=96.7±6.0 Intervention after: Mean=127.1±6.1 Control after: Mean=97.0±6.1	Anxiety Beck Anxiety Inventory (BAI; Beck et al., 1988) Burnout Maslach Burnout Inventory (MBI; Maslach and Jackson, 1981)	The higher the EI, the lower the emotional burnout and anxiety
Schinckus et al., 2018 Belgium	Cross-sectional study To Investigate if EI reduces diabetes-related distress and increases self-management behaviors and investigate if diabetes-related distress mediates the relationship between trait EI and diabetes self-management behaviors	Study 2: 146 adults. 80.5% females (mean age was 40±14years) Diabetes was treated mainly by insulin injections only (61%) or combined with oral medication (14.4%); 10.3% took oral medication without insulin and 14.4% had no medication for their diabetes but tried to reach/maintain a healthy lifestyle	Type 1 and 2 diabetes	Study 2: The Trait Emotional Intelligence Questionnaire (TEIQue-SF) Self-report mixed model Mean=4.81±0.828	Study 2: The Diabetes Self-Management Diabetes Self-Management Questionnaire (DSMQ; Schmitt et al., 2013) Diabetes Distress The Diabetes Distress Scale (DDS; Polonsky et al., 2005)	Diabetes-related distress acted as a mediator between EI and diabetes self-management. Furthermore, EI reduced diabetes-related distress, which improves self-management of the disease
Dehghan et al., 2014 Iran	Cross-sectional study To assess the relationship between attachment styles and EI and marital satisfaction in patients with Type 2 diabetes mellitus	200 married patients (mean age 43.92±12.46years) and 200 married healthy individuals (mean age 38.09±9.97years). Male gender, 51.0% vs. 50.0%. Not psychological or medical intervention described	Type 2 diabetes	The BarOn questionnaire (EQ-I) Self-report mixed model Diabetes group: Mean=313.61±22.00 Control group: Mean=322.08±30.19	Marital Satisfaction The ENRICH questionnaire (Fournier et al., 1983)	EI was positively related to marital satisfaction
Yalcin et al., 2008 Turkey	Randomized Controlled Trial and Cross-sectional study To investigate the relationship between EI and the health-related quality of life and well-being of individuals with Type 2 diabetes	36 adults. 50% females. Study group *n*=18 (mean age 54.33±7.34years) Control group *n*=18 (mean age 51.17±5.81years) No medical intervention described	Type 2 diabetes	The BarOn questionnaire (EQ-I) Self-report mixed model Intervention before: Mean=97.77±8.98 Control before: Mean=98.38±8.89 Intervention after: Mean=124.27±5.64 Control after: Mean=98.88±9.31	Quality of Life WHO-Quality of Life (WHOQOL-Bref; Skevington et al., 2004)	Positive relationship between EI and quality of life

#### Anxiety and Burnout

One study found a linked between EI and anxiety and burnout in adults with Type 2 diabetes. Karahan and Yalcin (2009) indicated that there was a negative relationship between these two variables; that is, the higher the EI, the lower the emotional burnout and anxiety.

#### Diabetes-Related Distress

One study was included in this section. Schinckus et al. (2018) showed that in adults with diabetes (Type 1 and 2 indistinguishable), diabetes-related distress acted as a mediator between EI and diabetes self-management. In addition, they found that EI reduced diabetes-related distress and that this improves self-management of the disease. This means that the higher the EI, the lesser the anxiety related to diabetes, which influences the implementation of more self-management behaviors.

#### Quality of Life and Well-Being

One study found a link between the EI and quality of life in adults with Type 2 diabetes. Yalcin et al. (2008) demonstrated a positive relationship between EI and quality of life; a higher EI level was related to a better quality of life and general well-being of people with diabetes.

#### Marital Satisfaction

Finally, marital satisfaction was a further variable analyzed in adults with Type 2 diabetes. Dehghan et al. (2014) showed that marital satisfaction was positively related to EI level; that is, people with diabetes who had a higher level of EI showed greater satisfaction with their relationship.

## Discussion

The present systematic review has focused on analyzing the relationship between EI and various biological and psychological variables in people with Type 1 and Type 2 diabetes to clarify the state of the art within the field and suggest future directions for research and intervention. We hypothesized that higher levels of EI would be related to better diabetes management and fewer emotional problems in this population. Given the variety of studies found in the literature search, we divided the results in four main sections.

In the first section, we included those studies in which the relationship between EI and HbA1c was analyzed. This was one of the main objectives of the review since HbA1c is a standard indicator of glycemic control. The results in both adults and adolescents showed that higher EI scores were related to lower HbA1c levels in people with Type 1 and 2 diabetes. This negative relationship was also found between the EI of the parents and the HbA1c of their adolescent offspring, except for a study in children, which found the opposite (and unexpected) result: a positive relationship between maternal EI and the HbA1c of their children. According to the authors, this result may be due to the use of a self-report which reflects the mothers’ subjective attitude toward their EI ability and also the fact that caregivers use ineffective cognitive strategies to regulate their emotions; that is, mothers may try to understand and control their emotions, but coping strategies may not be appropriate for responding to the diabetes challenges of their offspring (Cabello et al., 2021). From this, we can conclude that EI seems to play a relevant role in glycemic control. Diabetes (primarily Type 1 diabetes) has been related to anxiety, depression, and anxiety (Anderson et al., 2001; Grigsby et al., 2002; Nicolucci et al., 2013) which makes management of the disease more difficult. The results of this section might seem logical given that EI provides people with the resources required to manage their emotions and adequately face the challenges imposed, in this case, by the disease (Gratz and Roemer, 2004). In addition, these findings are consistent with the previous literature that offers evidence on the relationship between EI and physical and mental health (Martins et al., 2010; Fernández-Abascal and Martín-Díaz, 2015).

The second section included those studies evaluating the effect of EI training on people with Type 2 diabetes. The objective was to test whether EI training could improve various life aspects of people with diabetes, such as quality of life, anxiety, or glycemic control. The three studies showed that training in EI could improve all these variables compared to the control group of people with diabetes. Therefore, it is essential to note that training in EI can be a way to manage not only glycemic control but other psychological factors associated with the disease. The benefits of EI training have been found in the population with diabetes and healthy adults (Hodzic et al., 2018) and adolescents (Ruiz-Aranda et al., 2012). These studies showed EI training to be a plausible predictor of health by reducing risk factors such as anxiety, social stress, depression, or somatization and that the results can persist over time. The literature shows that EI dimensions can be better predictors of mental health than physical health (Fernández-Abascal and Martín-Díaz, 2015).

In the third section, EI levels were compared between people with Type 2 diabetes and a healthy population sample. The two studies showed that there were no statistically significant differences in EI between persons with diabetes and without diabetes. There is, however, no reason to expect individual differences as a consequence of the disease. Instead, we proposed that there might be individual differences in diabetes management according to EI levels, as shown in the first section. However, future lines of investigation should aim to analyze these results in people with Type 1 diabetes together with the employment of other EI models.

Finally, we included those studies in which EI was related to different variables of psychological adjustment and well-being and did not meet the criteria for the previous section. The results showed that EI was negatively related to diabetes-related distress, anxiety, and burnout and positively related to quality of life, well-being, and marital satisfaction. These results are consistent with the existing literature, which supports the hypothesis that emotional abilities are an essential factor for psychological adjustment that can mitigate both anxiety and depression in adolescents (Fernandez-Berrocal et al., 2006) and adults and improve their quality of life (Sánchez-Álvarez et al., 2016).

Taking together, the results of this review offer a starting point for a theoretical and practical understanding of the role played by EI in the management of diabetes. Specifically, in light of the studies shown, EI could be a protective factor for biological and psychological variables such as glycemic control, anxiety, and diabetes-related distress. Future investigations should also evaluate the role of EI as a mediator (and not just a mechanism) between the emotional state of individuals with Type 1 and 2 diabetes and biological outcomes such as HbA1c. More importantly, EI training seems to benefit both glycemic control and the psychological dimension of the patients. These results suggest that people with diabetes, that is, Type 2, will benefit from training in EI to maximize the strategies needed to deal with the disease on a daily basis.

Despite these preliminary conclusions, it is essential to highlight certain limitations that open possible future lines of investigation. First, relatively few articles have been published that study the role of EI in people with diabetes, since we found a total of 12. This implies that this research area is still emerging. Second, regarding the EI conceptualization employed in the different studies described, three instruments belonged to the performance-based ability models, three to self-report ability models, and seven to the self-report mixed models. It is crucial to consider which model is used to measure EI since, as previously shown, there is little correlation between them (Goldenberg et al., 2006), and they are not based on the same variables. Thus, the theoretical and clinical implications of the findings could differ according to the model employed. The model that has received the most empirical support is the performance-based ability model (Mayer et al., 2016), which was used in only three of the studies included in this review. In addition, the instrument based on the performance-based ability model only evaluates one of the four branches of the model (emotion perception), thus missing important information regarding the facilitating, understanding, and regulating EI branches. Future investigations should aim to confirm the results using instruments that cover all of the performance-based ability branches such as the MSCEIT (Mayer et al., 2002).

Thirdly, despite being different diseases, some studies do not differentiate between Type 1 and Type 2 diabetes. Both should be considered separately due to the different life implications of having one type of diabetes or another (Wong et al., 2020). Most studies included in the review have focused on Type 2, which usually appears in adulthood. In contrast, Type 1 diabetes frequently arises in childhood and adolescence. Adolescence is a developmental stage with which diabetes (as a chronic disease) interacts more negatively because the adolescent’s self-image is still developing. At this stage, interpersonal relationships become more important and a particular source of stress (Chamorro et al., 2002). In addition, the treatment of Type 1 diabetes adds a stressful condition due to daily blood glucose control, insulin dose adjustment, and the coping and management of hypo- and hyperglycemia, all of which disrupt the daily lives of people with diabetes (Ortiz Parada, 2006). Therefore, future research should analyze how EI improves lifestyles and reduce sources of stress in people with Type 1 diabetes, particularly during this developmental stage. Moreover, and considering the results of EI training in adults, we expect adolescents (who are particularly vulnerable) to benefit significantly from these interventions.

In conclusion, this review offers a starting point for proposing new research that could be beneficial for people with diabetes. At a theoretical level, the data suggest that EI could be a key protective variable for both psychological and biological adjustment in people with diabetes by supplying them with strategies for coping with the disease in daily life. Although further research is needed, the preliminary findings indicate that EI training could be an effective complementary tool for the management of diabetes in this population.

## Data Availability Statement

The original contributions presented in the study are included in the article/supplementary material, further inquiries can be directed to the corresponding author.

## Author Contributions

AP-F: conceptualization, data research, methodology, validation, investigation, writing – original draft preparation, and visualization. PF-B: term, conceptualization, validation, supervision, project administration, funding acquisition, and supervision. MG-C: term, conceptualization, data research, methodology, validation, writing – review and editing, visualization, and supervision. All authors contributed to the article and approved the submitted version.

## Funding

This work was supported by the Spanish Ministry of Economy, Industry, and Competitiveness (project: PSI2017-84170-R), Junta de Andalucía (UMA18-FEDERJA-114) and CTS-575.

## Conflict of Interest

The authors declare that the research was conducted in the absence of any commercial or financial relationships that could be construed as a potential conflict of interest.

## Publisher’s Note

All claims expressed in this article are solely those of the authors and do not necessarily represent those of their affiliated organizations, or those of the publisher, the editors and the reviewers. Any product that may be evaluated in this article, or claim that may be made by its manufacturer, is not guaranteed or endorsed by the publisher.
